# Undernutrition Affects Cell Survival, Oxidative Stress, Ca^2+^ Handling and Signaling Pathways in *Vas Deferens*, Crippling Reproductive Capacity

**DOI:** 10.1371/journal.pone.0069682

**Published:** 2013-07-26

**Authors:** Humberto Muzi-Filho, Camila G. P. Bezerra, Alessandro M. Souza, Leonardo C. Boldrini, Christina M. Takiya, Felipe L. Oliveira, Renata T. Nesi, Samuel S. Valença, Marcelo Einicker-Lamas, Adalberto Vieyra, Lucienne S. Lara, Valeria M. N. Cunha

**Affiliations:** 1 Institute of Biomedical Sciences, Federal University of Rio de Janeiro, Rio de Janeiro, Brazil; 2 Carlos Chagas Filho Institute of Biophysics, Federal University of Rio de Janeiro, Rio de Janeiro, Brazil; 3 National Institute of Science and Technology for Structural Biology and Bioimaging, Rio de Janeiro, Brazil; Paris Institute of Technology for Life, Food and Environmental Sciences, France

## Abstract

**Background:**

The aim of this work was to investigate the mechanisms by which chronic malnutrition (CM) affects *vas deferens* function, leading to compromised reproductive capacity. Previous studies have shown that maternal malnutrition affects the reproductive tracts of adult male offspring. However, little is known about the effects of CM, a widespread life-long condition that persists from conception throughout growth to adult life.

**Methodology/Principal Findings:**

Young adult male rats, which were chronically malnourished from weaning, presented decreased total and haploid cells in the *vas deferens*, hypertrophy of the muscle layer in the epididymal portion of the *vas deferens* and intense atrophy of the muscular coat in its prostatic portion. At a molecular level, the *vas deferens* tissue of CM rats exhibited a huge rise in lipid peroxidation and protein carbonylation, evidence of an accentuated increase in local reactive oxygen species levels. The kinetics of plasma membrane Ca^2+^-ATPase activity and its kinase-mediated phosphorylation by PKA and PKC in the *vas deferens* revealed malnutrition-induced modifications in velocity, Ca^2+^ affinity and regulation of Ca^2+^ handling proteins. The severely crippled content of the 12-kDa FK506 binding protein, which controls passive Ca^2+^ release from the sarco(endo) plasmic reticulum, revealed another target of malnutrition related to intracellular Ca^2+^ handling, with a potential effect on forward propulsion of sperm cells. As a possible compensatory response, malnutrition led to enhanced sarco(endo) plasmic reticulum Ca^2+^-ATPase activity, possibly caused by stimulatory PKA-mediated phosphorylation.

**Conclusions/Significance:**

The functional correlates of these cellular and molecular hallmarks of chronic malnutrition on the *vas deferens* were an accentuated reduction in fertility and fecundity.

## Introduction

It is generally accepted that reproductive performance in the adult is determined by a wide variety of influences, including nutritional status. It is clinically known that among the several forms of undernutrition, the life-long condition characterized by mild-to-moderate protein-energy malnutrition is the most common and is referred to as chronic malnutrition. This condition is defined by a deficiency of energy and nutrients over a period of several years that frequently impairs the growth and development of the child into adulthood [1,2]. Evidence that reproductive maturation and function are influenced by malnutrition is now emerging from animal studies and human populations [3–5]. It has been demonstrated that undernutrition during the fetal to pre-pubertal period is accompanied by changes in testicular structure with a consequent decrease in daily sperm production [5].

The *vas deferens*, located in the accessory male genitalia, is a paired organ that provides a link between the epididymis and prostate gland. It comprises circular and longitudinal smooth muscles with columnar epithelium lining the lumen [6]. In the ejaculatory process, contraction of the *vas deferens* is necessary for the transport of sperm through the ejaculatory duct to the urethra [6]. The presence in the *vas deferens* of molecular components responsible for Ca^2+^ transport and therefore for intracellular Ca^2+^ homeostasis, which is crucial for contraction, was demonstrated one decade ago [7]. Under conditions in which contraction is impaired – denervated or rapamycin-treated rat *vas deferens* – the sarco(endo) plasmic reticulum proteins are down-regulated [8,9], an event that could be associated with a decrease in reproductive capacity [6,10]. With respect to redox signaling, it has recently been demonstrated that reactive oxygen species (ROS) are implicated in male infertility [11]. For these reasons, *vasa deferentia* become an interesting model for evaluating oxidative stress, molecular alterations regarding Ca^2+^ transport and signaling pathways potentially linked to Ca^2+^ handling [12], and for correlating these to the loss of reproductive capacity.

The aim of this work was to determine the reproductive profile of adult male rats subjected to chronic malnutrition and the underlying adaptive consequences in *vas deferens* structure, Ca^2+^ homeostasis and tissue oxidative status. We have previously described the effects of protein kinases – which are effectors in signal cascades involved in the regulation of cellular Ca^2+^ handling [12] – and of ROS in different models of undernutrition [13–15]. In this study, we induced chronic malnutrition in male rats by using a diet that mimics the basic regional diet available in the vast sugarcane cultivation areas of Northeastern Brazil [16], which is a good model for chronic undernutrition worldwide. We demonstrated that chronic malnutrition compromises the reproductive profile in association with atrophy of the prostatic portion of the *vas deferens*, increased local oxidative stress and adaptive changes in intracellular Ca^2+^ handling, which are linked to protein kinase-mediated phosphorylation.

## Materials and Methods

### Ethical considerations

All procedures were performed in accordance with “The guide for care and use of laboratory animals’’ [DHHS Publication N^o^ (NIH) 85-23] and were also approved by the Committee for Experimental and Animal Ethics at the Federal University of Rio de Janeiro (protocol CEUA DFBCICB 007).

### Reagents and solutions

Recombinant 12-kDa FK506 (tacrolimus) binding protein (FKBP12), anti-sarco-endoplasmic reticulum Ca^2+^-ATPase type 2 (SERCA2), anti-protein kinase A (PKA) catalytic subunit and anti-protein kinase C (PKC) antibodies were purchased from Sigma-Aldrich. Anti-FKBP12, anti-plasma membrane Ca^2+^-ATPase (PMCA), anti-rabbit and anti-mouse immunoglobulin G (IgG) horseradish peroxidase-conjugated antibodies were provided by Santa Cruz Biotechnology. ^45^CaCl_2_ was purchased from GE Healthcare Biosciences. [^32^P]-inorganic phosphate (^32^P_i_) was obtained from the São Paulo Institute of Energetic and Nuclear Research. [γ-^32^P]-adenosine triphosphate ([γ-^32^P] ATP) was prepared as described by Maia and coworkers [17]. All solutions were prepared with deionized glass-distilled water.

### Diet

The deficient diet entitled Regional Basic Diet (RBD) [16] contained (in g/g%) meat fat (0.0035), jerked meat (3.7), sweet potato (12.8), beans (18.3) and manioc flour (64.8), which were cooked, pulverized and mixed with filtered water to form a solid wet mass. The mixture was shaped into small squares that were dehydrated at 60°C for 24 h. The contents of main dietary nutrients were as previously described [13,15,16]. It is worth mentioning the original statement by Teodósio et al. [16], as a synthesis of experimental and epidemiological studies that allows a rat diet and metabolism to be correlated with human ones. *“…RBD produces, in the rat, a type of malnutrition similar to that prevalent among children from Northeast Brazil: impairment of growth, as in the nutritional dwarfism associated to other clinical signs of marasmus. Identical basic food patterns have been found in 38 countries of the Near and the Middle East, Central America and Peru, where some clinical signs similar to those seen in RBD induced malnutrition have been described. Our findings lead to the conclusion that an experimental model like this can provide valuable information for a better knowledge of malnutrition in countries where nutritional and socioeconomic conditions are similar to ours.”*

### Animals

Male Wistar rats were weaned at 21 days of age and reared three per cage with free access to food and water in a room maintained at 25 ± 1^o^C with a 12 h light–dark cycle. Twenty-four rats were equally divided into two main experimental groups: (i) control (CTRL), in which the animals were given a standard rat chow (Labina); and (ii) chronically malnourished (CM), in which the animals were fed with RBD from weaning until 90 days of age. After analysis of the reproductive parameters *in vivo*, these two main groups were each divided into three subgroups for the study of different parameters: (i) cell counting in testis and *vas deferens* (n = 10 in CTRL; n = 7 in CM); (ii) histological evaluation of the *vas deferens* (n = 6); and (iii) determination of the *vas deferens*/body weight ratio followed by the preparation of *vas deferens* homogenates, which were used for the biochemical assays detailed below (n = 6).

### Reproductive profile analyses

**Figure 1:**
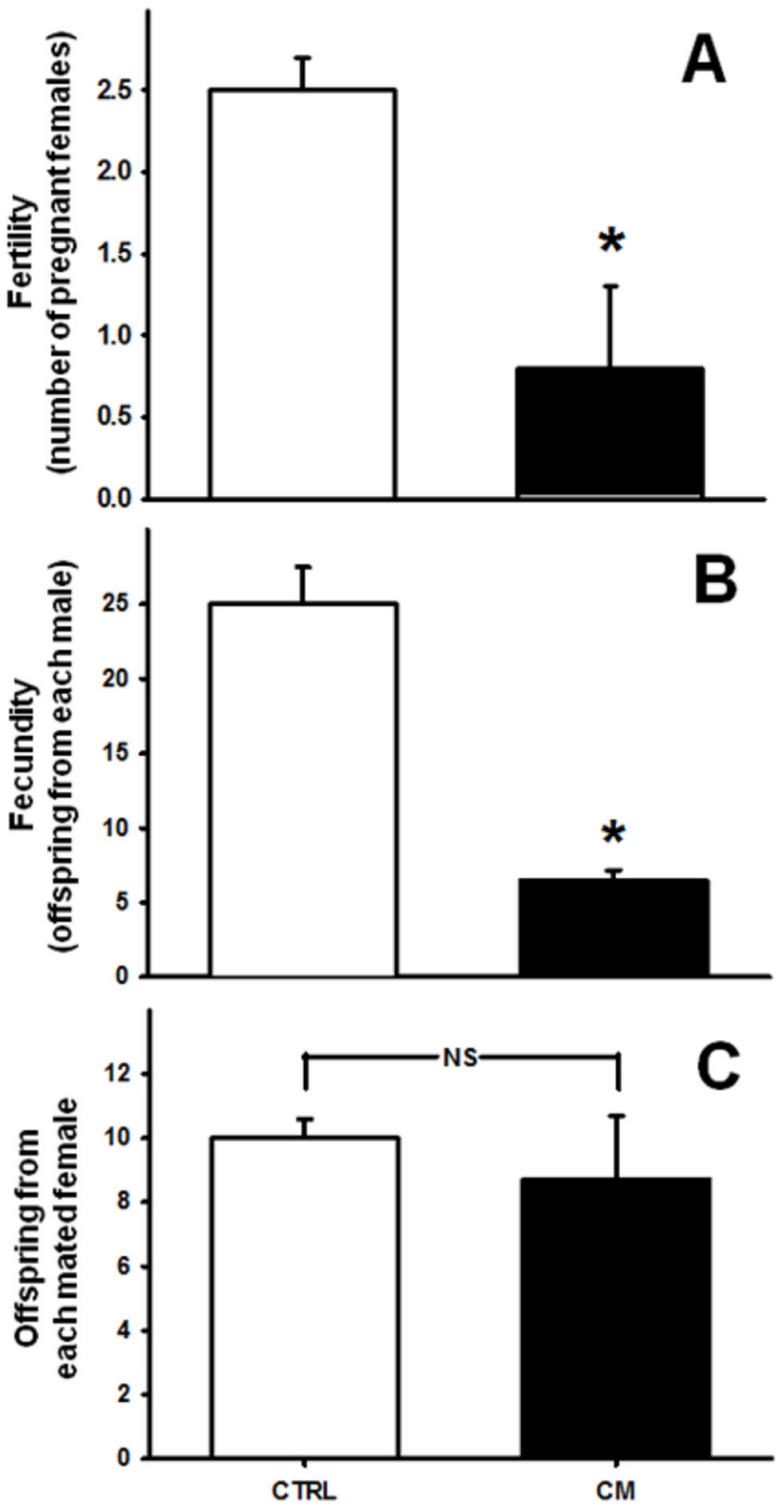
Chronic malnutrition-induced alterations in the reproductive profile of adult male rats. The animal groups were well-nourished rats (CTRL) and chronically malnourished rats (CM), as indicated on the *abscissa*. (A) Fertility: number of well-nourished pregnant females resulting from mating with one well-nourished (empty bar) or one chronically malnourished (black bar) male. Data are represented as mean ± SEM of matings from six groups (1 male with 3 females each) of CTRL and CM rats. (B) Fecundity: offspring from each CTRL or CM male rat. Data are represented as mean ± SEM of offspring from six mated groups. (C) Offspring from each mated female. Results are mean ± SEM from 18 dams. *P<0.05 *vs.* the corresponding CTRL (unpaired Student’s *t*-test); NS: no statistical difference.

Young adult virgin male rats from the CTRL and CM groups were mated by the harem method (one male and three normally nourished females) and housed in polypropylene cages (43 cm × 30 cm × 15 cm) for 10 days, aiming to cover at least two periods of estrus, in a biotherium maintained at 25 ± 1^o^C with a 12 h light−dark cycle (light cycle starting at 07:00 h a.m). The average age of the female rats was 90 days and all were virgins. It is worth mentioning that no female rats were subjected to chronic malnutrition and that the offspring number from each mated female was the same, indicating that the female rats were healthy (see below Figure 1C). The animals had free access to standard chow and drinking water. Careful twice-daily observations by one member of the authors’ team, which has wide experience of handling rats in a biotherium, confirmed that the receptiveness of males and females was similar in the two experimental groups. In addition, observations at random during the lighting cycle demonstrated no impairment in the movements of undernourished males that are preludes to copulation, in the few episodes that were noted. In a period between 21 and 34 days after mating the following parameters were evaluated: fertility (number of females that became pregnant after mating with one male rat), fecundity (offspring from each male), and offspring from each mated female. These data allowed us to evaluate changes in the reproductive profile of the malnourished animals.

### Total and haploid cell counts

Sperm cells were counted according to Joyce and coworkers [18] with slight modifications, and DNA was stained using the technique of Vindelov and coworkers [19]. Briefly, testis, epididymis and *vas deferens* (n = 10, CTRL and n = 7, CM, from different animals) were suspended in saline solution containing 150 mM NaCl and 0.05% (v/v) Triton X-100 (280 mg wet whole tissue/ml solution) and carefully disrupted using a Potter-Elvejhem homogenizer with a Teflon pestle. Subsequently, the cell suspensions were incubated in 1 ml of a Vindelov solution containing 0.1% (v/v) Na-citrate buffer, 0.1% (v/v) Triton X-100, 0.5 g/ml RNAse and 50 µg/ml propidium iodide (Sigma-Aldrich). From this last mixture 1 ml was used for counting; the data were corrected for the dilution and they were finally expressed as counts × s^-1^ per 100 mg of wet whole tissue (for each organ). After 15–20 min of incubation, the viable cells were quantified and the DNA content was analyzed by flow cytometry (FACSCalibur Flow Cytometer, Becton Dickinson). Cell viability was evaluated by the exclusion of Trypan blue in a Neubauer chamber. The cell dissociation procedure did not affect fluorescence under the experimental conditions used in this study. The total cell number was obtained from the number of events per 10 s of acquisition using the specific software Cell-Quest (BD Biosciences). DNA content was evaluated and the cell cycle was established as follows: cells with fragmented DNA (<*n*), haploid in G0-G1 phase (*n*), S phase (>*n* but <2*n*) and diploid in G2/M phase (2*n*). The samples were acquired and analyzed using CellQuest and WinMDI 2.9, respectively (both from BD Biosciences).

### Histological analyses

The animals were perfused with sterile saline containing heparin (10 U/ml) *via* the left cardiac ventricle followed by 4% (w/v) phosphate-buffered paraformaldehyde (PFA) (pH 7.4). The *vasa deferentia* were carefully removed, dissected and transversally sectioned in their epididymal (initial first quarter) and prostatic (final quarter) portions and incubated in PFA solution for 24 h. These portions were dehydrated in graded ethanol solutions (70–95%) and embedded in paraffin. Sections 5 µm thick were stained with Masson’s trichrome for histological studies. The images were captured using a digital camera (Evolution, Media Cybernetics Inc.) coupled to a light microscope (Eclipse 400). Thirty high quality images (2048 × 1536 pixels buffer) were captured using Pro Plus 4.5.1 software (Media Cybernetics), as previously described [20]. All the quantifications were done by a single observer. The thickness of the muscular coat area (μm^2^) was calculated by the difference between the total *vas deferens* area and the lumen area. There were six animals in each experimental group.

### Preparation of total homogenate from *vas deferens* tissue

*Vasa deferentia* were removed and immersed in a cold solution (4^o^C) containing 137 mM NaCl, 2.7 mM KCl, 11.9 mM NaHCO_3_, 0.36 mM Na^+^-phosphate, 5.5 mM glucose, 1.8 mM CaCl_2_ and 0.4 mM MgCl_2_, equilibrated with a carbogen gas mixture (95% O_2_/ 5% CO_2_) (pH 7.4) at room temperature. The lumen of the tissue was washed three times with 1–3 ml of this solution prior to homogenization (2,500 rpm), which was carried out using an Ultraturrax disperser (IKA Works, Inc.) -20,500 rpm for 60 s followed by two steps of 20,500 rpm for 30 s with a 20 s interval between the steps – in a medium containing 250 mM sucrose, 5.5 mM Tris-HCl (pH 7.4), 2 mM dithiothreitol, 0.2 mM phenylmethylsulfonyl fluoride, 2 µg/ml antipain, 5 µg/ml aprotinin and 1 mM EDTA. Repeated and careful flushing of the *vas deferens* lumen allowed sperm cells to be removed completely in studies directed to the tissue. The total homogenate was centrifuged at 108,000 × *g* for 60 min; the pellet was resuspended in 250 mM sucrose and stored under liquid N_2_. The protein content was determined by the Folin reagent method [21].

### Lipid peroxidation, protein carbonylation and total free sulphydryl groups in *vas deferens* tissue

After removal of the *vas deferens*, lipid peroxidation was assessed by measuring the levels of thiobarbituric acid reactive species (TBARS) according to the Buege & Aust method [22] with slight modifications [15]. Briefly, the organs were homogenized in 5 ml 1.15% KCl per gram in an ice-bath and then 1 ml 0.375% (w/v) thiobarbituric acid in 15% (w/v) trichloroacetic acid (TCA) was added per milliliter of tissue suspension. The samples were heated in a water-bath at 100^°^C for 15 min and then cooled. The tubes were centrifuged for 10 min at 5000 rpm, the supernatants were collected and the absorbance was measured at 535 nm. The results were expressed in μmol malondialdehyde / mg of the original *vas deferens* mass. Protein carbonylation was assayed according to Menegali and coworkers [23] with slight modifications. Total *vas deferens* homogenates (prepared as described above) were used to measure protein carbonyl content by evaluating the amount of labeled protein-hydrazone derivatives obtained with 2,4-dinitrophenylhydrazine (DNPH). These derivatives were extracted with 10% (v/v) trichloroacetic acid (TCA), followed by treatment with a 1:1 (v/v) mixture of ethanol/ethyl acetate and re-extraction with 10% TCA. The resulting precipitate was dissolved in 6 M guanidine. The difference in absorbance at 370 nm between this solution and a solution without 2,4-dinitrophenylhydrazine was used to calculate the amount of 2,4-dinitrophenylhydrazine incorporated (nmol carbonyl / mg of *vas deferens* protein). The total free sulphydryl group content (−SH) was measured following the method described by Ellman [24] with the modifications introduced by Herken and coworkers [25] using the same preparations as those for the protein carbonylation assays, and the results were expressed as μmol total free sulphydryl groups / mg of *vas deferens* protein.

### Measurement of Ca^2+^-ATPase activity in homogenized *vas deferens* tissue

Homogenate fractions (80 µg protein) were incubated for different times (0.5–150 min) or for a fixed time (120 min) in a medium (0.25 ml, 37^o^C) containing 50 mM MOPs-Tris (pH 7.4), 10 mM NaN_3_, 0.3 mM EGTA, 5 mM [γ-^32^P] ATP (disodium salt; specific activity ~1.5 × 10^10^ Bq/mmol), 4 mM MgCl_2_ and 60 mM KCl, in the presence or absence of 3 µM thapsigargin. Enough CaCl_2_ was added to give free Ca^2+^ concentrations in the range 1 nM–10 μM (or a fixed 10 µM concentration), calculated according to Sorenson et al. [26]. Thapsigargin-sensitive Ca^2+^-ATPase activity (related to sarco(endo) plasmic reticulum Ca^2+^-ATPase – SERCA) was determined by the difference between the Ca^2+^-ATPase activity measured in the absence (total Ca^2+^-ATPase) and presence of thapsigargin. Reactions were stopped by adding 1 ml cold 26% (w/v) charcoal in 0.1 N HCl. The tubes were centrifuged at 1,500 × *g* for 15 min at 4^o^C, and 0.25 ml of supernatant containing the released ^32^P_i_ was counted by liquid scintillation. The results were expressed as nmol P_i_ released / mg of protein (time course experiments) or nmol P_i_ released / mg protein at 2 h (Ca^2+^ concentration dependence experiments).

### SDS-PAGE and Western blotting of *vas deferens* tissue proteins

SDS-PAGE was carried out according to Laemmli [27]. The homogenate samples (80 µg) were separated and transferred to nitrocellulose membranes. For FKBP12 content analysis, human recombinant FKBP12 (2 µg) was used as a positive control. The blotted membranes were blocked for 60 min in 5% non-fat milk containing 0.1% Tween 20, incubated overnight with specific primary antibodies and probed for 60 min with an anti-rabbit or anti-mouse IgG horseradish peroxidase-conjugated antibody, as described in the figure legends. Immunoreactivity was detected using enhanced chemiluminescence by exposure of the membranes to Hyperfilm-ECL. Since β-actin content was lower in the *vas deferens* from the undernourished groups, protein loading was controlled using Ponceau red staining. The results are presented as PMCA, SERCA2 or FKBP12 content in homogenized *vas deferens* tissue, taking the content in the *vas deferens* of control rats as 100%.

### Determination of protein kinase activity in *vas deferens* tissue homogenates

The protein kinase activities were analyzed in homogenized *vas deferens* tissue by measuring the incorporation of the γ-phosphoryl group of [γ-^32^P] ATP into histone in the absence or presence of the specific cyclic AMP-dependent protein kinase (PKA) and protein kinase C (PKC) inhibitors, 10 nM PKAi_5-24_ and 10 nM calphostin C, respectively, as previously described [15,28]. The reaction was started by adding [γ-^32^P] ATP (10 µM; specific activity ~1.5 × 10^11^ Bq/mmol) to a medium (0.1 ml) containing 20 mM HEPES-Tris (pH 7.0), 4 mM MgCl_2_, 1.5 mg/mL histone and 0.7 mg/ml protein. After 5 min, the reaction was stopped by adding 0.1 ml of 40% (w/v) TCA and the samples were immediately placed on ice. After vigorous stirring, an aliquot of 0.1 ml was removed, filtered through a Millipore filter (0.45 µm pore size) and successively washed with ice-cold 20% (w/v) TCA and 0.1 M phosphate buffer (pH 7.0). The radioactivity was quantified by liquid scintillation. The results were expressed as nmol of P ~ histone incorporated per mg of *vas deferens* tissue protein at 5 min (for both the PKA and the PKC activities).

### Quantification of the phosphorylation levels of the 140 (PMCA) and 110 KDa (SERCA) bands in *vas deferens* tissue

Kinase-mediated phosphorylation of Ca^2+^-ATPase in homogenized *vas deferens* tissue was determined as previously described [28,29]. The membranes (1 mg/ml) were preincubated for 10 min at 37^o^C in a reaction medium containing 50 mM Hepes-Tris (pH 7.4), 0.5 mM ouabain, 5 mM MgCl_2_, 10 mM NaN_3_, 10 mM NaF, 0.3 mM EGTA, 0.34 mM CaCl_2_ (10 µM free Ca^2+^) and 1.1 M hydroxylamine in the absence of protein kinase inhibitors, or in the presence of either 10 nM PKAi_5-24_ (to block PKA activity) or 10 nM calphostin C (to ensure inhibition of the diacylglycerol-dependent PKC isoforms). The phosphorylation reaction was started by adding a mixture of 5 mM [γ-^32^P] ATP (specific activity ~3.0 × 10^11^ Bq/mmol) and 120 mM KCl (final concentrations). Ten min later the reaction was stopped by adding 50 µl of sample buffer [27], and the proteins were separated by SDS-PAGE (6% bis-acrylamide/acrylamide). The gel protein bands were transferred to nitrocellulose membranes and exposed for 48 h to a phosphor screen and analyzed using a PhosphorImager Storm 860 (Molecular Dynamics) to measure the intensity of the [^32^P]-phosphorylated 140 kDa (PMCA) and 110 kDa (SERCA) bands recognized by their specific primary antibodies. Specific phosphorylation of Ca^2+^-ATPase by PKA or PKC was quantified by the difference between band intensities in the absence and presence of the respective inhibitors, and normalized by the intensities of the same bands immunodetected using the corresponding antibodies. The results were expressed as the ratio between the ^32^P signals from the autoradiograms (at 140 or 110 kDa), corrected by the PMCA and SERCA2 contents in the corresponding bands of the same nitrocellulose membrane. For both PKA- or PKC-mediated regulatory phosphorylations of PMCA and SERCA, the levels obtained with control rats were taken as 100%.

### Statistical analysis

The data are presented as means ± S.E.M. Differences between the parameters studied in the CTRL and CM groups were analyzed using unpaired Student’s *t*-tests. P<0.05 was considered the criterion for statistical significance.

## Results

### Chronic malnutrition alters the reproductive profile of adult male rats

Figure 1A shows that fewer normonourished females became pregnant after mating with CM male rats (0.8 ± 0.5) than with CTRL male rats (2.5 ± 0.2). Moreover, the females generated fewer total offspring derived from CM male rats (6.5 ± 0.7) than from CTRL male rats (25.0 ± 2.5) (Figure 1B). The offspring were the same regardless of whether the females became pregnant after mating with CTRL or CM males (Figure 1C), showing that the observed impairment is exclusively related to the CM male rats.

### Evaluation of total and haploid cells in testis, epididymis and *vas deferens* of CTRL and CM male rats

**Figure 2:**
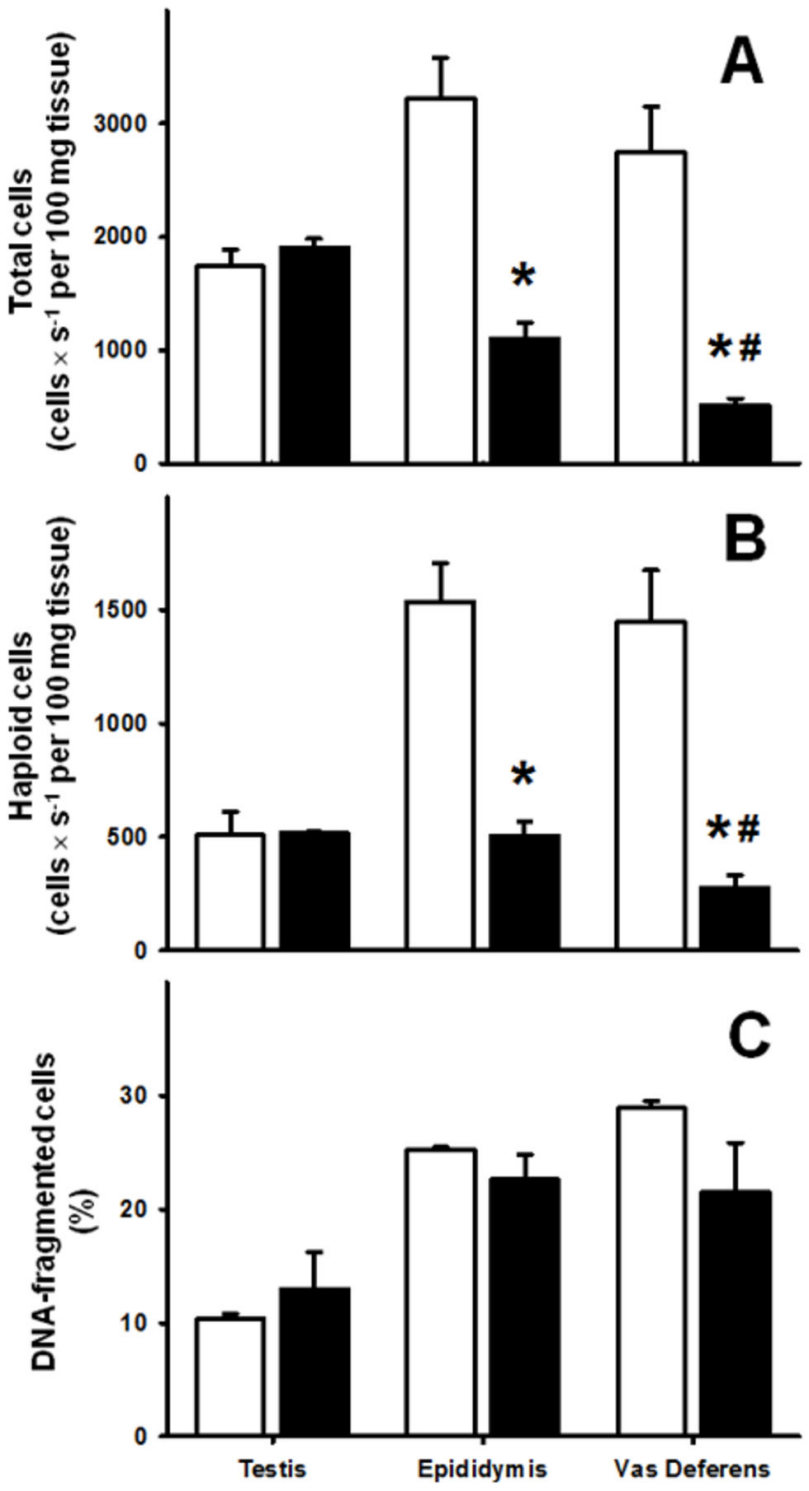
Germ cell counts in testis, epididymis and *vas deferens*. Total (A) and haploid (B) cells were counted in testis, epididymis and *vas deferens* from different CTRL (n = 10) and CM (n = 7) rats. (C) Percentage of DNA-fragmented cells in the total cell populations of the three organs. Results are mean ± SEM. *P<0.05 *vs.* the respective CTRL; ^#^ P<0.05 *vs.* cell counts in epididymis of CM rats (unpaired Student’s *t*-test).

To evaluate whether the impairment of reproductive capacity in chronically malnourished male rats is related to alterations in the production and transport of reproductive cells, the numbers of total, haploid and DNA-fragmented cells in testis, epididymis and *vas deferens* were counted (Figure 2). Cytometric analysis demonstrated (in number of cells × s^-1^ per 100 mg of wet whole tissue) that chronic malnutrition did not alter the total number of cells in the testis. However, the total cell counts were reduced by ~65% and ~80%, respectively, in epididymis and *vas deferens* (Figure 2A). Moreover, chronic malnutrition provoked a reduction in the number of haploid cells in the epididymis (from 1,550 to 500 cells × s^-1^ per 100 mg of wet tissue) and *vas deferens* (from 1,450 to 260 cells × s^-1^ per 100 mg of wet tissue) (Figure 2B). The proportions of DNA-fragmented cells in testis, epididymis and *vas deferens* were not affected in the undernourished group (Figure 2C).

At first sight it is odd that there are similar numbers of haploid cells in epididymis and *vas deferens*, because the cauda epididymis and not the *vas deferens* is considered to be the storage compartment for haploid spermatozoa [30]. However, it should be noticed that we used whole epididymis and not cauda epididymis and that counting was normalized per 100 mg of wet tissue. Since the epididymis mass is about four times the total *vas deferens* mass (Table 1), the total number of haploid cells in the first organ is actually higher than in the *vas deferens*.

**Table 1:** Body weight, organ (testis, epididymis and *vas deferens*) weight and organ/body weight ratio of CTRL male and CM male rats.

Parameter	CTRL (n = 6)	CM (n = 6)
Body weight (g)	409 ± 7	170 ± 3*
Testis weight (mg)	1,683 ± 56	1,642 ± 193
Epididymis weight (mg)	411 ± 12	279 ± 38*
*Vas deferens* weight (mg)	104 ± 2	57 ± 1*
Testis weight / body weight (mg/g)	4.11 ± 0.14	9.66 ± 1.14*
Epididymis weight / body weight (mg/g)	1.00 ± 0.03	1.64 ± 0.22*
*Vas deferens* weight / body weight (mg/g)	0.26 ± 0.07	0.34 ± 0.08*

* P<0.05 with respect to the corresponding CTRL, as assessed by an unpaired Student’s *t*-test.

### Chronic malnutrition promotes changes in *vas deferens* tissue mass and architecture

Table 1 demonstrates that the CM male rats at the age of 90 days presented with a significantly lower epididymis and *vas deferens* weight than the CTRL group. The testis weight did not differ between the two groups. Owing to the pronounced decrease in body weight of the CM rats, the corresponding indexes (masses of the organs corrected by body weight) were considerably higher in the undernourished group (Table 1).

**Figure 3:**
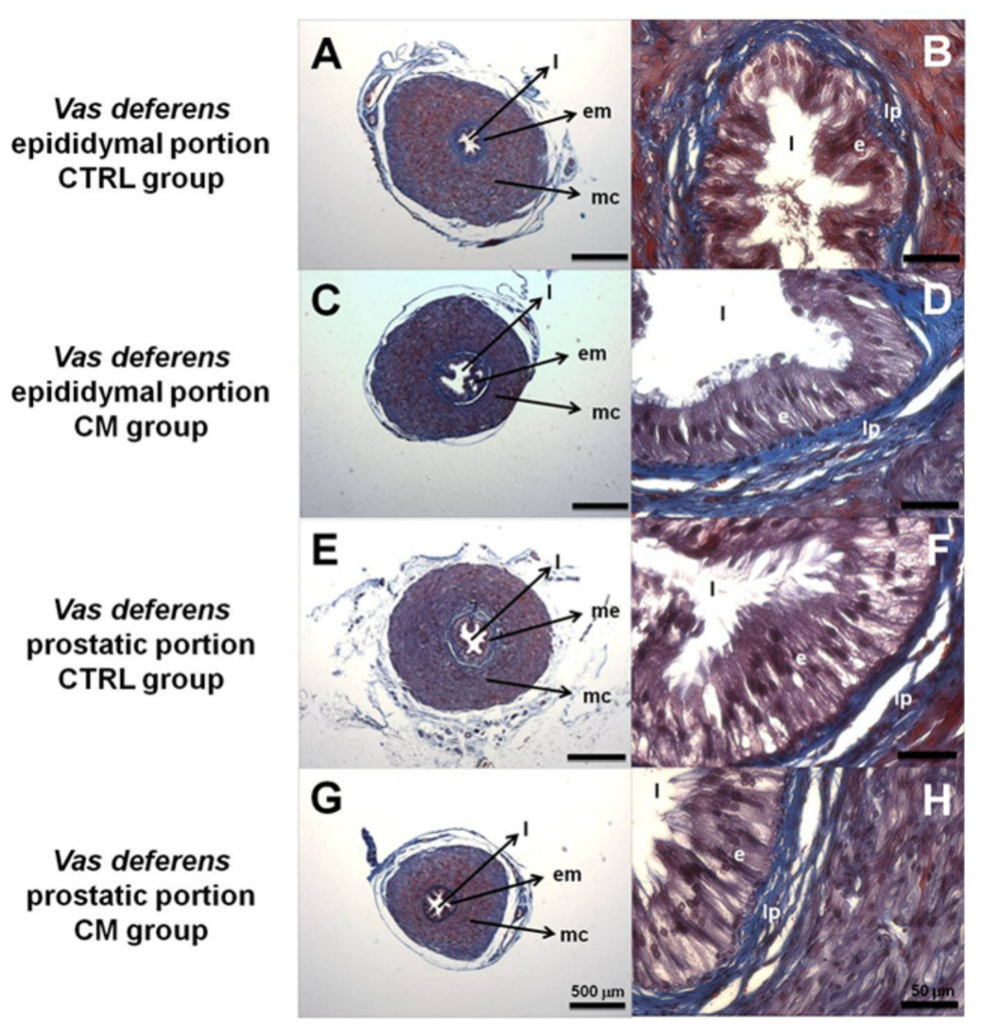
Representative photomicrographs (Masson’s Trichrome) of *vas deferens* tissue. The epididymal (A–D) and prostatic (E–H) portions of *vas deferens* from CTRL and CM rats are indicated in the legends on the left side of the panels. Key: In the middle low magnification panels: l, lumen; em, epithelial mucosa; mc, muscular coat. In the right high magnification panels: l, lumen; e, epithelium; lp, lamina propria. The panels are representative figures of four histological analyses in different fields using six different male rats in each group.

To determine the degree to which the *vas deferens* architecture was compromised in the chronically malnourished male rats, the organs were dissected and transversally sectioned in their epididymal and prostatic portions and stained with Masson’s trichrome. Figure 3 illustrates the lumen (l), the epithelial mucosa (em) and the muscular coat (mc) at lower magnification (panels A, C, E and G). Histomorphometric analyses demonstrated that the muscular coat area was greater in the epididymal portion and smaller in the prostatic portion of the CM group than CTRL (Table 2). At higher magnification (panels B, D, F and H) no changes were detected in the villi, in the high-columnar cell shape of the epithelium (e) or in the thickness of the lamina propria (lp).

**Table 2:** Muscular coat area of the two portions of the *vas deferens* in CTRL male and CM rats, obtained from histomorphometric analysis of the images presented in Figure 3 (panels A, C, E and G).

	Muscular coat area (µm^2^)	
*Vas deferens* portion	CTRL (n = 6)	CM (n = 6)
Epididymal	4769 ± 15	5779 ± 274*
Prostatic	13382 ± 516	6689 ± 272*

* P<0.05 with respect to the corresponding CTRL, as assessed by an unpaired Student’s *t*-test.

The organs were cut at the first quarter of the epididymal portion and at the final quarter of the prostatic portion.

### Chronic malnutrition causes cellular oxidative damage in *vas deferens* tissue

**Figure 4:**
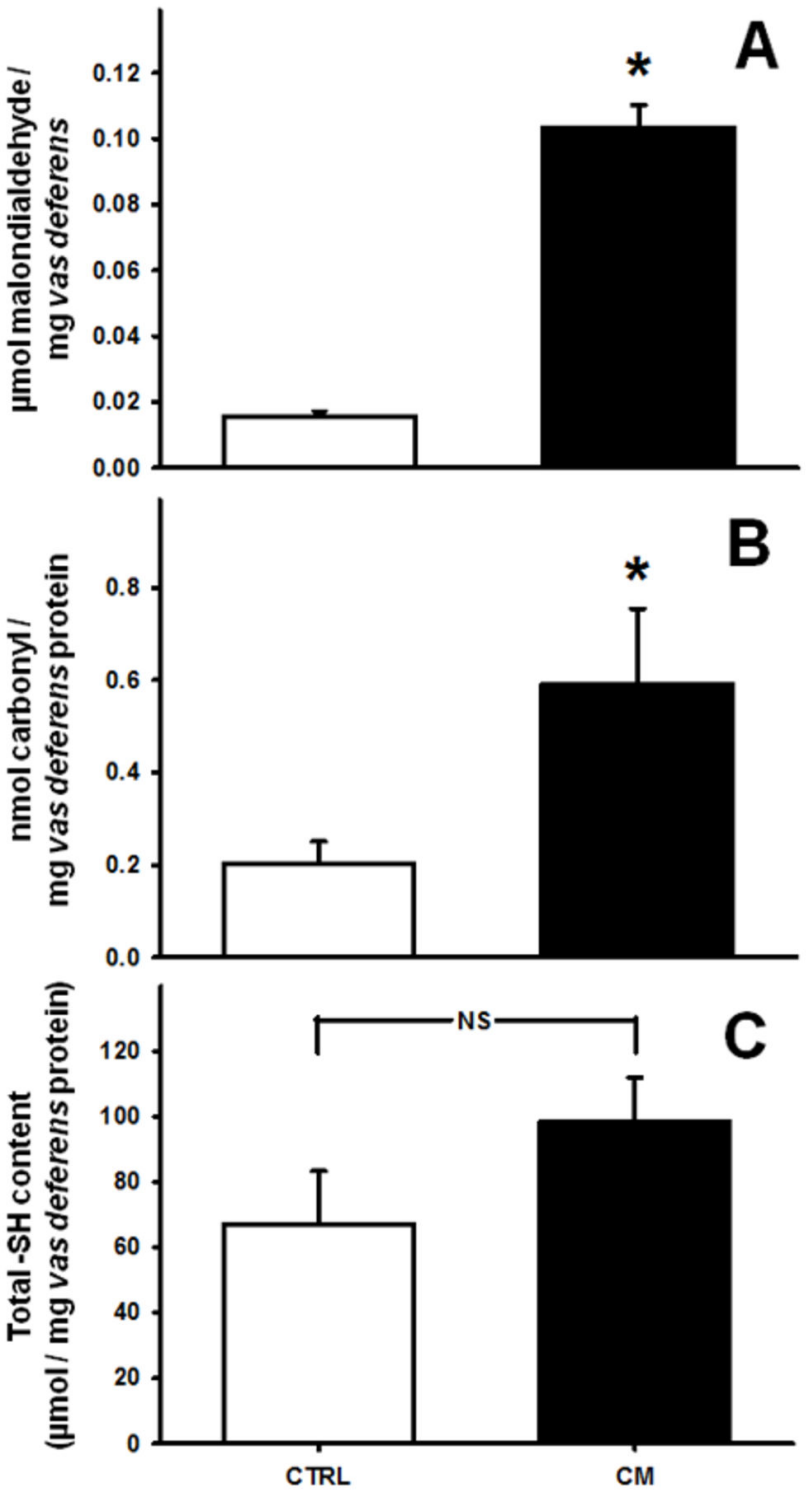
Oxidative damage caused by chronic malnutrition in *vas deferens* tissue. Levels of lipid peroxidation (A), protein carbonylation (B) and total free sulphydryl group content (C) in the *vas deferens* tissue cells of CTRL (empty bars) and CM (black bars) groups. Results are expressed as mean ± SEM. (A) and (C) were determined using three animals from each group; in (B), six rats from each group were used for determinations. *P<0.05 *vs.* CTRL. NS: no statistical difference.

Figure 4 illustrates the oxidative damage in the *vas deferens* caused by chronic malnutrition. In CM male rats, lipid peroxidation in whole homogenates (estimated by the level of thiobarbituric acid reactive species) was seven-fold higher than in CTRL (Figure 4A). Oxidative damage was also evidenced by a three-fold increase in protein carbonylation (Figure 4B); the total amount of free −SH was similar in the CTRL and CM groups (Figure 4C).

### Alterations in Ca^2+^ handling in *vas deferens* tissue during chronic malnutrition involve the content, activity and regulation of Ca^2+^-transporting proteins

The presence of the molecular entities responsible for Ca^2+^ homeostasis was determined in the *vas deferens* tissue one decade ago [7]. Here we investigated the effect of chronic malnutrition on the activities of Ca^2+^ pumps: the thapsigargin-resistant Ca^2+^-ATPase (plasma membrane Ca^2+^-ATPase, PMCA) and the thapsigargin-sensitive Ca^2+^-ATPase (sarco-endoplasmic reticulum Ca^2+^-ATPase, SERCA).

**Figure 5:**
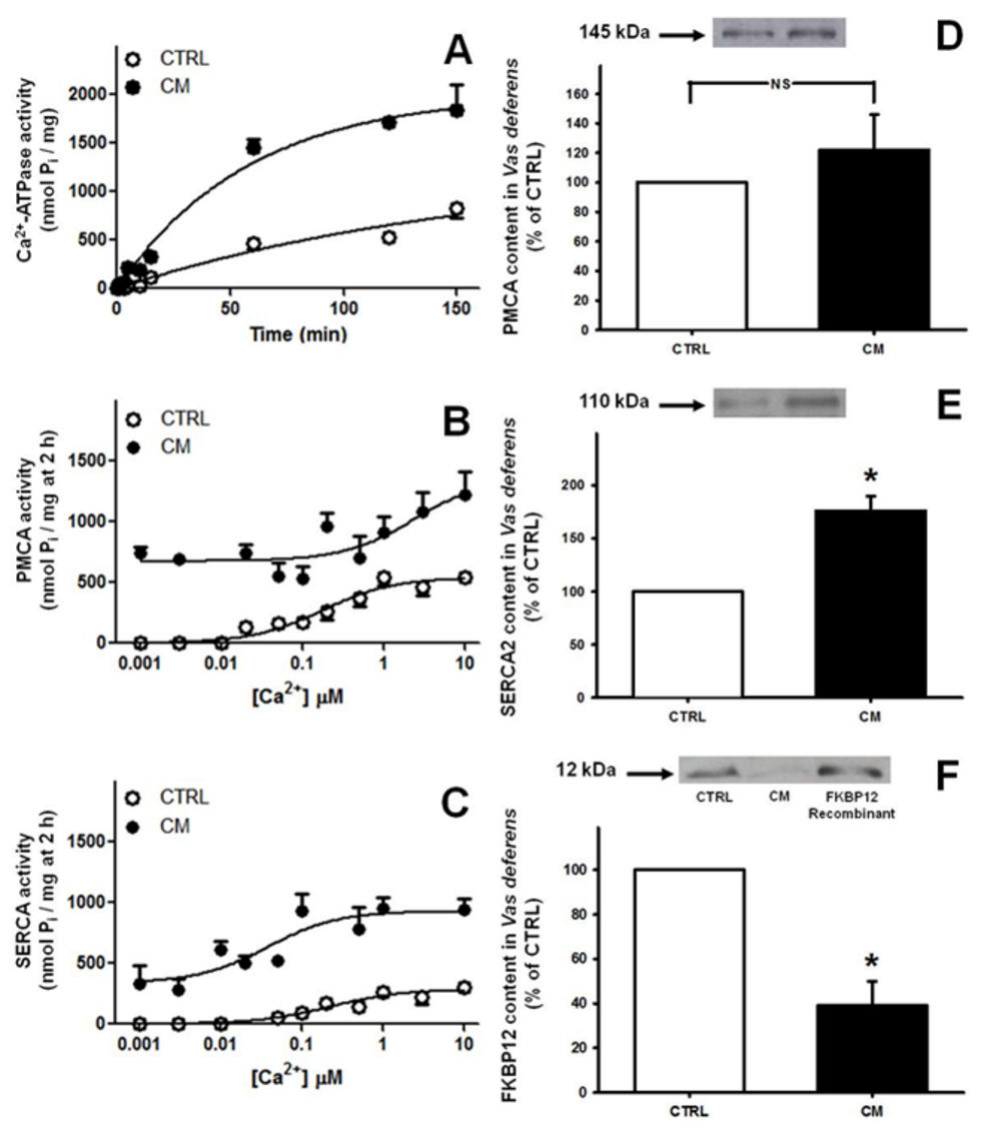
Ca^2+^-ATPase activity and content of Ca^2+^-handling proteins in whole homogenate of *vas* *deferens* tissue. (A) Time course of Ca^2+^-ATPase activity. The Ca^2+^-ATPase activity was assayed at the different times of incubation presented on the *abscissa*, starting at 30 s, using the CTRL (empty circles) and CM (filled circles) groups. Results are expressed as mean ± SEM (n = 5). The smooth curves were fitted to the experimental points using the equation P_i*, t*_ = P_i*, max*_ (1 - e^*-k t*^), where *k* is the first-order rate constant and P_*i, max*_ is the asymptotic value of P_i_ release. (B) and (C) Ca^2+^ concentration dependence of the Ca^2+^-ATPase activities in whole homogenate of *vas deferens*. Values were obtained for PMCA (B) and SERCA (C) activities of CTRL (empty circles) and CM (filled circles) groups. Results are expressed as mean ± SEM (n = 5). The smooth lines were adjusted to the experimental points by non-linear regression using the equations: v = V_max_ × [Ca^2+^]/(K_0.5 Ca_ + [Ca^2+^]) or v = v_c_ + V_max_ × [Ca^2+^]/(K_0.5 Ca_ + [Ca^2+^]) for the CTRL and CM groups, respectively. V_max_ is the maximal hydrolysis rate, and K_0.5 Ca_ is the free Ca^2+^ concentration required for half-maximal activation of the CTRL activities and also represents the free Ca^2+^ concentration required for half-maximal activation of the lower affinity components (above 0.01 µM) of PMCA and SERCA in the CM group. The term v_c_ represents the very high affinity component of the Ca^2+^ curve in CM. (D) (E) and (F) Contents of PMCA, SERCA2 and FKBP12 in *vas deferens*. Ca^2+^ pumps PMCA and SERCA2 and immunophilin FKBP12 were immunodetected in whole homogenates of *vas deferens* from the CTRL (empty bars) and CM (black bars) groups. (D) PMCA. Upper panel: representative immunoblotting of three determinations. Lower panel: graphic representation of three immunoanalyses of different preparations. Samples from CTRL and CM were analyzed in parallel on the same gel; densitometric values were corrected for protein loading using Ponceau red and are presented as percentage of CTRL. (E) SERCA2. Upper panel: representative immunoblotting of three determinations. Lower panel: graphic representation of three immunoanalyses of different preparations. Samples were analyzed as described in (D). (F) FKBP12. Upper panel: representative immunoblotting of three determinations from different rats from the CTRL and CM groups, each of which was analyzed using recombinant FKPB12 as a parallel positive control, as shown in immunoblotting legend. Lower panel: graphic representation of three immunoanalyses of *vas deferens* FKPB12. *P<0.05 *vs*. the corresponding CTRL; NS: no statistical difference.

The time course of total Ca^2+^-ATPase activity demonstrated increased overall active Ca^2+^ handling across the membranes of the CM rats, which was quantified in the following experiments. Figure 5A demonstrates that total Ca^2+^-ATPase activity – i.e. one way to evaluate ATP-dependent Ca^2+^ transport – can be described as a first-order process in both experimental groups, thus allowing the rate constant *k* to be measured (see legend to Figure 5). The *k* values were 0.006 min^-1^ and 0.017 min^-1^ in CTRL and CM respectively, demonstrating that the global ATP-dependent pumping activity in *vas deferens* is three-fold higher in malnourished rats.

This activity was dissected using thapsigargin (a specific SERCA inhibitor) to evaluate the contributions of PMCA and SERCA, and their respective kinetic parameters were investigated in assays carried out at different free Ca^2+^ concentrations (Figure 5B and 5C). The hyperactivity of total Ca^2+^-ATPase in the CM group seen in the time course experiments was confirmed in the Ca^2+^ concentration dependence assays, where an additional very high affinity component in the nanomolar range became evident. This component, which was saturated at Ca^2+^ concentrations below 0.01 µM, appeared in both PMCA (Figure 5B) and SERCA (Figure 5C). The kinetic parameters (see legend to Figure 5) are summarized in Table 3.

**Table 3:** Kinetic parameters: Ca^2+^ concentration dependence of PMCA and SERCA from CTRL and CM preparations of *vas deferens* (n = 5).

Ca^2+^-ATPase		v_c_ (nmol P_i_/mg at 2 h)	K_0.5 Ca_ (μM)	V_max_ (nmol P_i_/mg at 2 h)
*PMCA*				
	CTRL	−	0.17	538
	CM	675	2.27^1^	686^1^
*SERCA*				
	CTRL	−	0.22	283
	CM	334	0.04^1^	595^1^

^1^ Parameters corresponding to the lower affinity component of the Ca^2+^ curve.

The effect of chronic malnutrition on PMCA and SERCA2 content was also evaluated. PMCA content was not altered in the chronically malnourished rats (Figure 5D), despite the higher activity of this enzyme. However, SERCA2 content was ~80% higher in the CM than the CTRL group (Figure 5E). FKBP12 content was ~60% lower in the *vas deferens* of CM rats (Figure 5F).

### Chronic malnutrition increases protein kinase activity in the *vas deferens* tissue

**Figure 6:**
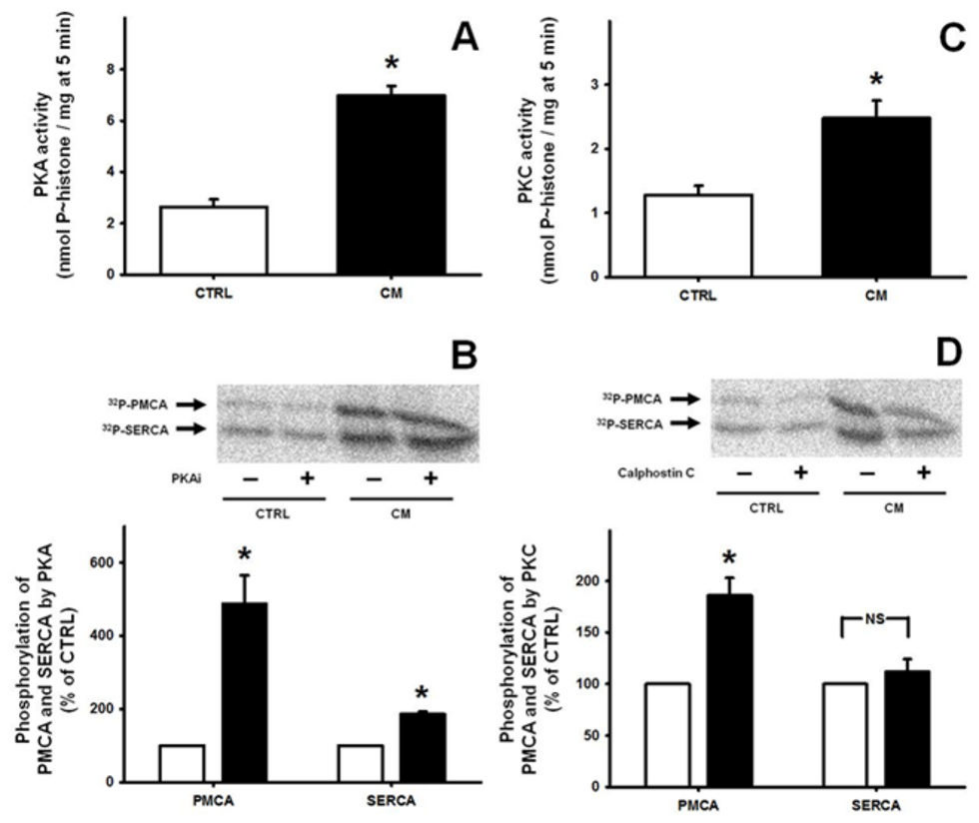
PKA and PKC activities and PKA- and PKC-mediated regulatory phosphorylations of PMCA and SERCA from *vas deferens* tissue. (A) Cyclic AMP-dependent protein kinase (PKA) activity in *vas deferens* homogenates from four CTRL (empty bar) and four CM (black bar) preparations. Results are mean ± SEM. (B) Upper panel: representative hydroxylamine-resistant (kinase-mediated) autoradiograms of ^32^P-phosphorylations of *vas deferens* PMCA (^32^P-PMCA) and SERCA (^32^P-SERCA), as indicated by arrows on the left side of the panel. Assays were carried out in the absence (−) or presence (+) of 10 nM PKAi, the specific PKA inhibitor, as shown on the corresponding *abscissa*. Lower panel; graphic representation of PKAi-sensitive ^32^P-phosphorylations in *vas deferens* from three different preparations from CTRL (empty bars) and CM (black bars) rats. The densitometric values were corrected by simultaneous immunodetection of each Ca^2+^-ATPase, which are shown in the Western blots of Figure 5 (D and E). Each bar corresponds to the difference between phosphorylations in the absence and presence of PKAi, and values for each Ca^2+^-ATPase are expressed as percentages of the corresponding CTRL. The SEM were calculated from absolute values and converted to percentage values. *P<0.05 vs. the corresponding CTRL. (C) Calphostin-sensitive protein kinase C (PKC) activity in *vas deferens* homogenates from four CTRL (empty bar) and four CM (black bar) preparations. Results are mean ± SEM. (D) Upper panel: representative hydroxylamine-resistant (kinase-mediated) autoradiograms of ^32^P-phosphorylations of *vas deferens* PMCA (^32^P-PMCA) and SERCA (^32^P-SERCA), as indicated by arrows on the left side of the panel. Assays were carried out in the absence (−) or presence (+) of 10 nM calphostin C, an inhibitor of both classical and novel isoforms of PKC, as shown on the corresponding *abscissa*. Lower panel: graphic representation of calphostin C-sensitive ^32^P-phosphorylations carried out with *vas deferens* from three different preparations from CTRL (empty bars) and CM (black bars) rats. The densitometric values were also corrected by immunodetection of the ATPases, as described above for PKA. Each bar corresponds to the difference between phosphorylations in the absence and presence of calphostin C, and values are presented as percentages of the corresponding CTRL. The SEM were calculated from absolute values and converted to percentages. *P<0.05 vs. the respective CTRL. NS: no statistical difference.

Regulatory kinase-mediated phosphorylations – especially those catalyzed by PKA and PKC – play a crucial role in the regulation of Ca^2+^ transporters in non-excitable and excitable cells [31,32]. Thus, the influence of chronic malnutrition on PKA and PKC in *vas deferens* tissue was determined. The more than 170% increase in PKA activity in the CM group (Figure 6A) was accompanied by 400% and 100% rises in the hydroxylamine-resistant (regulatory) PKAi-sensitive phosphorylation of PMCA and SERCA, respectively (autoradiograms in Figure 6B). PKC activity was 100% higher in the *vas deferens* of CM rats (Figure 6C), matching a similar increase in calphostin-sensitive PKC-mediated PMCA phosphorylation, without influence on the basal PKC-mediated phosphorylation of SERCA (autoradiograms in Figure 6D). Western blotting demonstrated that chronic undernutrition did not modify the content of PKA or of four PKC isoforms (α, ε, λ, and ξ), which are representative of the three PKC families [33] (not shown).

## Discussion

Compromised reproductive capacity is considered an adaptive mechanism by which animals improve their chances of individual survival during famine by deferring reproduction until more calories are available [3]. In the present study we demonstrated that the mechanisms by which chronic malnutrition impairs fertility and fecundity (Figure 1), reducing the number of haploid cells without modification in the number of DNA-fragmented cells (Figure 2), are likely to be related to an increase in local oxidative stress (Figure 4), dystrophic alterations of the muscular coat (Figure 3 and Table 2) and modifications of Ca^2+^-handling proteins (Figure 5). Regarding the last-named processes, kinase-mediated regulatory phosphorylations appear to play a crucial role (Figure 6). Since the numbers of DNA-fragmented cells were the same in testis, epididymis and *vas deferens*, it is plausible that apoptosis in the germ line stands at the same levels in both groups.

The reduced reproductive capability of CM adult male rats could be due, at least in part, to the decreased content of total and haploid cells. It is likely that the loss of the smooth muscular coat in the prostatic portion of the *vas deferens*, together with the alterations in the Ca^2+^ handling machinery and in its linked regulatory pathways, might compromise *vas deferens* function, ultimately leading to a decrease of total and haploid cells. These altered mechanisms affecting cell propulsion from the testis, where the number of total and haploid cells is the same (Figure 2), could culminate in cell death in a tissue environment where oxidative stress reaches high levels, as demonstrated by the intense lipid peroxidation and protein carbonylation (Figure 4). Previous studies revealed that increased generation of reactive oxygen species leads to compromised male reproductive capacity [34], and increased oxidative stress seems to be a hallmark of undernutrition status under different conditions and is associated with alterations in protein kinases as we previously demonstrated [13–15].

There are no previous observations relating chronic malnutrition to alterations in spermatogenesis. Thus, the results showing reduction of haploid cell numbers in the testis and epididymis indicate that chronic malnutrition globally affects the reproductive tract, similarly to perinatal malnutrition, which is accompanied by changes in testicular structure with a consequent decrease in daily sperm production [5]. Since we observed no modifications in the receptiveness of males and females or in the movements of males that usually precede copulation – despite their reduced size – we can conclude that the quantity and quality of ejaculate are dominant over other possible factors in a process culminating in reduced fertility. Changes in the number and quality of sperm, including important morphological alterations, were described in 90 days-aged male rats that were undernourished by perinatal protein restriction [35]. However, even a small contribution from the additional factors mentioned above could have enhanced a process that culminated in decreased fertility.

One should also consider the possibility of undernutrition-provoked alterations in the endocrine activity of the testis in terms of systemic and testicular androgen levels. This possibility seems unlikely for two reasons. First, testis weight is preserved in the CM group. Second, undernutrition caused by a deficient diet with a similar protein content given in the very sensitive window of placental life does not affect the serum or intratesticular concentrations of testosterone, follicle-stimulating hormone or luteinizing hormone [35]. In chronically malnourished animals, maintenance of hormonal levels could represent the strategy for preservation of reproduction and species perpetuation, despite the challenge imposed by undernutrition.

The cell counts showed that chronic malnutrition affects the *vas deferens* more severely than the epididymis (Figure 2A and 2B), and for this reason we emphasized the study of this organ. The opposite malnutrition-induced modifications in the muscle coat in the epididymal and prostatic regions of the *vas deferens* could also contribute to the reduced number of haploid cells. Hypertrophy of the former presumably increases the delivery of cells to the prostatic portion, whose reduced muscle thickness would probably lead to a lowered forward delivery of cells. Exposure of the cells to the unfavorable environment discussed above would be likely to contribute to their decrease in the *vas deferens* lumen. The epididymal and prostatic parts of the *vas deferens* have different contractile properties and their synchrony is crucial for an adequate forward movement of cells along the reproductive tract [36]. This synchrony seems to be disrupted by chronic malnutrition, becoming an important adjuvant for oxidative stress-induced cell damage (Figure 4A).

The observations that free −SH remained unchanged (Figure 4C), implying that there was no non-specific oxidation of cysteine residues, and that the epithelial architecture was maintained, favor the hypothesis that malnutrition affects specific molecular targets in the *vas deferens*. These targets certainly include the active Ca^2+^ transporters, which are subject to oxidative attack in processes associated with elevated levels of carbonylated proteins [37], as in the case encountered here (Figure 4B). It is of interest that carbonyl compounds have well-documented, specific effects on the male genital tract and testis of rats [38] and for that reason deserved special attention in this study, even though they only give a partial view of the redox state of a tissue. Furthermore, it is possible that besides the alterations in intracellular Ca^2+^ handling, which will be discussed below, the excitation/contraction process in the *vas deferens* is also affected by ROS, like the level of sympathetic neurotransmission in chronically diabetic rats, where energy metabolism is also compromised [39].

The alterations in thickness of the muscle coat of the *vas deferens* in malnourished rats are accompanied by profound modifications in the kinetics and regulation of the ensemble of molecules responsible for Ca^2+^ homeostasis in smooth muscle [40]. The increased Ca^2+^-ATPase activities and the mirror alterations in Ca^2+^ affinities of PMCA and SERCA (Figure 5A–5C Table 3) together with the altered PMCA, SERCA2 and FKBP12 contents (Figure 5D–5F) allows us to infer that there is a deregulated interaction of Ca^2+^ with the contractile machinery of *vas deferens* cells. Two hypotheses can be proposed regarding these modifications. First, it is likely that the increased pumping activities derive from leakiness induced by the oxidative stress that leads to increased cytosolic free Ca^2+^; second, the alterations in contents of the three proteins in the tissue cells possibly reflect specific qualitative alterations in amino acid content, besides limitations of energy availability. In view of these observations it can be proposed that an ensemble of malnutrition-induced Ca^2+^ transport alterations combine so as to impair the adequate handling of this ion by the contractile machinery of the muscle layer of *vas deferens* tissue cells. In this regard, the huge increase in PKA-mediated phosphorylation of PMCA in the CM group compared to the lesser increase in PKC-mediated phosphorylation (Figure 6B and 6D) could account for this imbalance. We demonstrated that PKA is a key activator of PMCA in the innervated faces of electrocytes from *Electrophorus* *electricus* L. [31] and in renal cells [28,29], whereas PKC acts as an inhibitor [41], and the undernutrition-induced imbalance between these two kinases could account for an altered Ca^2+^ handling. To date, it has not been demonstrated that SERCA is a substrate for PKA. However, a serine residue (Ser^38^) in the SERCA2 amino acid sequence is phosphorylated by Ca^2+^/calmodulin-dependent protein kinase (CaM kinase). This phosphorylation induces an increase in the V_max_ of Ca^2+^ transport [42], suggesting the possibility of indirect activation of SERCA by PKA in the *vas deferens* tissue *via* a pathway in which CaM kinase participates. However, further studies regarding Ca^2+^ concentrations in muscle cells of the *vas deferens* and physiological motility of the whole organ in chronically malnourished rats are needed to elucidate the detailed mechanisms.

In conclusion, the findings described here provide experimental evidence regarding the molecular mechanisms involved in the compromised *vas deferens* function provoked by chronic malnutrition. Therefore, they convey an advance in the understanding of how this systemic disorder can negatively affect male reproductive capacity in populations worldwide.
